# Reversible inhibition of lysine specific demethylase 1 is a novel anti-tumor strategy for poorly differentiated endometrial carcinoma

**DOI:** 10.1186/1471-2407-14-752

**Published:** 2014-10-09

**Authors:** Emily R Theisen, Snehal Gajiwala, Jared Bearss, Venkataswamy Sorna, Sunil Sharma, Margit Janat-Amsbury

**Affiliations:** Center for Investigational Therapeutics (CIT) at Huntsman Cancer Institute, University of Utah, Salt Lake City, UT USA; Department of Pharmaceutics and Pharmaceutical Chemistry, College of Pharmacy, University of Utah, Salt Lake City, UT USA; Division of Medical Oncology, University of Utah School of Medicine, Salt Lake City, UT USA; Department of Obstetrics and Gynecology, Division of Gynecologic Oncology, University of Utah, Salt Lake City, UT 84132 USA; Center for Nanomedicine, Nano Institute of Utah, Salt Lake City, UT USA

## Abstract

**Background:**

Endometrial cancer is the most common gynecologic malignancy. Type II endometrial carcinoma is often poorly differentiated and patients diagnosed with Type II disease (~11%) are disproportionately represented in annual endometrial cancer deaths (48%). Recent genomic studies highlight mutations in chromatin regulators as drivers in Type II endometrial carcinoma tumorigenesis, suggesting the use of epigenetic targeted therapies could provide clinical benefit to these patients. We investigated the anti-tumor efficacy of the LSD1 inhibitor HCI2509 in two poorly differentiated Type II endometrial cancer cell lines AN3CA and KLE.

**Methods:**

The effects of HCI2509 on viability, proliferation, anchorage-independent growth, global histone methylation, LSD1 target gene induction, cell cycle, caspase activation and TUNEL were assayed. KLE cells were used in an orthotopic xenograft model to assess the anti-tumor activity of HCI2509.

**Results:**

Both AN3CA and KLE cells were sensitive to HCI2509 treatment with IC_50_s near 500 nM for cell viability. Inhibition of LSD1 with HCI2509 caused decreased proliferation and anchorage independent growth in soft agar, elevated global histone methylation, and perturbed the cell cycle in both cell lines. These effects were largely dose-dependent. HCI2509 treatment also caused apoptotic cell death. Orthotopic implantation of KLE cells resulted in slow-growing and diffuse tumors throughout the abdomen. Tumor burden was distributed log-normally. Treatment with HCI2509 resulted 5/9 tumor regressions such that treatment and regressions were significantly associated (p = 0.034).

**Conclusions:**

Our findings demonstrate the anti-cancer properties of the LSD1 inhibitor HCI2509 on poorly differentiated endometrial carcinoma cell lines, AN3CA and KLE. HCI2509 showed single-agent efficacy in orthotopic xenograft studies. Continued studies are needed to preclinically validate LSD1 inhibition as a therapeutic strategy for endometrial carcinoma.

**Electronic supplementary material:**

The online version of this article (doi:10.1186/1471-2407-14-752) contains supplementary material, which is available to authorized users.

## Background

Endometrial carcinoma (EC) arises from the lining of the uterus and is the most commonly diagnosed invasive gynecologic malignancy, exceeding the incidence of cervical, ovarian, vaginal, and vulvar cancers combined [1, 2]. With 50,230 new cases and 8,590 deaths estimated in the U.S. for 2014 it is the fourth most prevalent cancer among women in developed countries, and the sixth worldwide [1, 3, 4]. Most patients present with low-grade early-stage disease, but patients diagnosed with more aggressive, high-grade, advanced disease that has spread beyond the uterus will progress within 1 year [5]. EC has been broadly classified into two subtypes based on differing clinico-pathologic characteristics. Over 80% of ECs are categorized as Type I endometroid adenocarcinomas [6, 7], while the remaining are Type II serous, clear-cell, poorly differentiated, and grade 3 endometrioid carcinomas [6, 7]. Type I malignancies are associated with extended periods of elevated estrogen exposure, obesity, and estrogen and progesterone receptor positivity. These cancers present and are diagnosed in earlier stages and are typically more differentiated, responsive to progesterone treatment, and consequently have a more favorable prognosis [6, 7]. Type I tumors are more common than Type II tumors in pre- and perimenopausal women [6]. On the other hand, Type II EC more frequently occurs in postmenopausal women and tumors are typically poorly differentiated [7]. Unlike Type I, Type II disease is unrelated to hyperestrogenic risk factors, diagnosed in later stages of the disease, and is clinically more aggressive. While representing only ~15% of all clinical cases Type II disease is responsible for around ~48% of endometrial cancer-related deaths, despite adjuvant chemotherapy and radiation, mainly due to metastasis and recurrent disease [7]. Better therapeutic strategies are needed for these patients.

No single hereditary risk factor plays a dominant role in endometrial cancer, which is driven by an interplay of genetic, environmental, and epigenetic factors. Several instances of epigenetic misregulation have been described in endometrial cancer. Specifically, alterations in DNA methylation have been broadly observed, with promoter hypermethylation leading to silencing of the progesterone receptor and other tumor suppressors like *MLH1*, *APC*, *MGMT*, and *PTEN* [8, 9]. Hypomethylation at the *CD133* promoter has been observed in tumor initiating cells, suggesting epigenetic regulation does affect the mechanisms driving tumorigenicity and disease recurrence [10]. Additionally, the expression of various histone modifying enzymes are altered in endometrial cancer, including histone deacetylases as well as the histone methyltranferase *EZH2.* Their inhibition decreases proliferation and invasiveness in endometrial cancer cell lines [11–14]. Importantly, the advent of next generation sequencing has allowed further characterization of the molecular etiology of Type II EC, shedding more light on possible epigenetic targets and allowing for novel treatment options to be developed. Analysis of the genomic landscape of Type II EC identified somatic mutations in members of the nucleosome remodeling and deacetylase complex (NuRD), *CHD4* and *MBD3*, as well as mutations in the chromatin and transcriptional regulators *EP300*, *ARID1A*, and *TAF1* as candidate driver events [15–17]. While the functional significance of these mutations in Type II EC remains to be elucidated, these data underscore the significance of the interplay between genetic and epigenetic factors in the development, progression and prognosis of Type II EC.

Unlike genetic mutations, epigenetic changes, including DNA methylation and posttranslational modifications of histones, are dynamic and reversible through pharmacological intervention, such that the readers, writers, and erasers of epigenetic marks are emerging therapeutic targets [18, 19]. Patterns of histone lysine methylation are maintained in a more cell-type specific manner than DNA methylation or histone acetylation, and it is thought that pharmacologically modulating offending histone lysine methyltransferases or demethylases can confer increased therapeutic specificity and decreased dose-limiting off-target toxicities [20–23]. Lysine-specific demethylase 1 (LSD1) is a histone lysine demethylase with specificity for mono- and dimethylated histone H3 lysine 4 (H3K4) and lysine 9 (H3K9) [24, 25]. Methylation at H3K4 is generally considered to be permissive, while H3K9 methylation is repressive [26]. LSD1 is upregulated in several malignancies and associated with decreased differentiation, aggressive tumor biology, and poor prognosis [27–34]. HCI2509 is a small molecule inhibitor of LSD1 that has shown *in vitro* anti-tumor efficacy in triple negative breast cancer, and single-agent *in vivo* efficacy in both Ewing sarcoma and castration-resistant prostate cancer [35–38]. A cell line panel showed one Type II EC cell line, AN3CA, to be sensitive to treatment with HCI2509 [35]. In this investigation, we validate this result in another Type II cell line, KLE, and further evaluate the mechanism of action by testing whether HCI2509 causes global changes in histone methylation, modulates the LSD1 target gene *HMOX1* and *CDH1*, and disrupts oncogenic transformation. More importantly, we also assess whether HCI2509 displays any anti-tumor efficacy *in vivo*. In order to most accurately represent disease spread mimicking human EC as well as more predictable therapeutic efficacy, we utilize an orthotopic xenograft mouse model to demonstrate the *in vivo* activity of HCI2509 against poorly differentiated Type II EC.

## Methods

### Antibodies and reagents

Immunodetection was performed with the following antibodies: anti-α-Tubulin (Calbiochem CP06), anti-LSD1 (Cell Signaling C69G12), anti-H3 (Cell Signaling Technology D2B12), anti-H3K4me3 (Cell Signaling Technology C42D8), anti-H3K9me2 (Cell Signaling Technology 9753), anti-H3K27me3 (Cell Signaling Technology C36B11). Propidium iodide (Sigma P4864), medroxyprogesterone 17-acetate (MPA; Sigma M1629). HCI2509 is previously described [35].

### Cell culture, proliferation, colony formation assays, cell viability, and caspase 3/7 activation

Endometrial carcinoma cell lines AN3CA and KLE were obtained from ATCC and maintained in the DMEM/F12 supplemented with 10% FBS, 100 units/ml penicillin, and 100 μg/ml streptomycin. All experiments were performed prior to passage 10. Proliferation assays (3T5) and colony formation assays were performed as previously described [39, 40]. Cell viability and caspase activation were performed using Cell Titer-Glo and Caspase 3/7-Glo (Promega). The same vehicle (0.3% DMSO) was used for both HCI2509 and MPA in all *in vitro* treatments.

### Western blots and quantitative reverse-transcriptase polymerase chain reaction (qRT-PCR)

AN3CA and KLE cells were seeded in triplicate in 6-well dishes at a density of 3.5 × 10^5^ cells/well or 2 × 10^5^ cells/well, respectively. Cells were treated with varying concentrations of HCI2509 for 48 hours, harvested, and flash frozen for protein or RNA extraction. Total RNA was extracted from treated cells using an RNeasy Plus kit (Qiagen). cDNA was generated using qScript cDNA SuperMix (Quanta Bioscience). Template was then amplified, detected, and quantified using SYBR green fluorescence. Each replicate was normalized to the internal housekeeping gene (RPL19) and induction was calculated relative to the vehicle control. The following primers were used: RPL19_fwd 5′-ATGTATCACAGCCTGTACCTG-3′, RPL19_rev 5′-TTCTTGGTCTCTTCCTCCTTG-3′; HMOX1_fwd 5′-AACTTTCAGAAGGGCCAGGT-3′, HMOX1_rev 5′-GTAGACAGGGGCGAAGACTG-3′; CDH1_fwd 5′-TGCCCAGAAAATGAAAAAGG-3′, CDH1_rev 5′-GTGTATGTGGCAATGCGTTC-3′.

### Cell cycle analysis

1 × 10^6^ cells (KLE, AN3CA) were seeded in 10 cm dishes and treated with either vehicle alone or HCI2509 for the appropriate duration, trypsinized, centrifuged at 1000 rcf for 5 min, and fixed in ice cold 70% ethanol. Staining was performed by centrifuging 1.5 × 10^6^ fixed cells at 770 rcf for 5 minutes, aspirating ethanol, and resuspending in 350 μL of staining buffer (4 mM citrate, 3% PEG8000, 50 μg/mL propidium iodide (PI), 180 units/mL RNase, 0.1% Triton X-100) incubating at 37°C for 20 minutes, and adding 350 μL of salting buffer (400 mM NaCl, 3% PEG8000, 50 μg/mL PI, 0.1% Triton X-100). Cells were analyzed on a BD FACSCanto with Software Diva vs6.1.3 (BD Biosciences San Jose CA).

### TUNEL and fluorescence microscopy

9 × 10^4^ AN3CA cells or 3 × 10^4^ KLE cells were seeded onto glass coverslips in a 12-well dish. Cells were treated with either vehicle or 3 X EC50 HCI2509 for 72 hours to correlate with the caspase activation assay. Cells were fixed in formalin and stained with the DeadEnd Fluorescent TUNEL system (Promega). DNase treatment and no labeling reaction were used as positive and negative internal controls, respectively. Cells were then stained with AlexaFluor Phalloidin (1:100) (Molecular Probes) and DAPI (0.3 μM) (Molecular Probes). Fluorescent cell images were collected on a Zeiss Axioskop2 mot plus microscope with a 40X dry objective (NA 0.75 NeoFluor), Axiocam MR camera, and Axiovision v4.8.1 software (Carl Zeiss MicroImaging, Inc.).

### *In vivo*xenograft studies

All xenograft experiments were performed in accordance with protocol 11–12001 approved by the University of Utah IACUC. Female nude mice (strain J:Nu) were purchased from Jackson Laboratory (Bar Harbor, ME) and housed under appropriate conditions. Mice were anesthetized with 100 mg/kg ketamine and 10 mg/kg xylazine and surgical procedures were carried out in a clean room on a circulating water warming pad set to 38°C. A frontal midline incision was made to enter the peritoneal cavity and 2 × 10^6^ KLE cells expressing luciferase were implanted into the bifurcation of the uterus in 50 μL of 1:1 DMEM/F12:Matrigel (Corning). Following tumor cell implantation, the peritoneum and skin were each sutured separately and recovery was assessed daily for 7 days by weight measurements and visual inspection. VivoGlo Luciferin (Promega) was resuspended in PBS at a concentration of 30 mg/ml and passed through a 0.22 μM filter. Mice were imaged on day 7 using an IVIS Spectrum (PerkinElmer). Images were acquired 10 minutes after intraperitoneal (IP) administration of 100 μL luciferin. Mice with detectable tumor on day 7 were randomized into three groups: Vehicle only (n = 7; 100 μL 1:1 PBS:PEG400 IP daily), HCI2509 30 mg/kg (n = 9; 100 μL suspension IP daily), or untreated (n = 3). Body weight was tracked three times per week and luminescence was tracked weekly for the entire treatment period of 35 days. At day 42 of the study, mice were sacrificed, organs including uteri harvested and weighed, and fixed in formalin prior to paraffin embedding.

## Results

### HCI2509 impairs viability, proliferation, and transformation in Type II endometrial cancer cell lines

**Figure 1:**
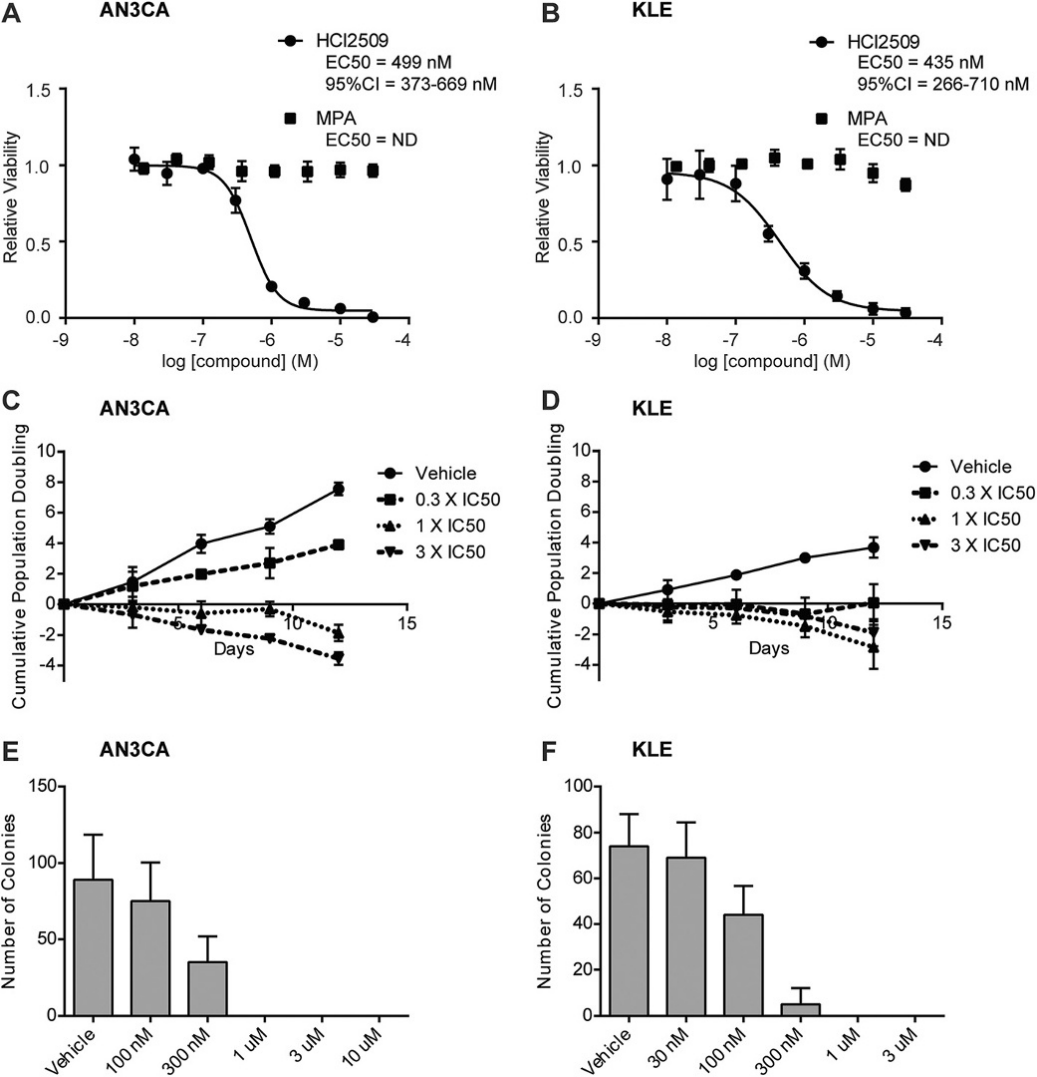
**HCI2509 impairs cell viability, proliferation and transformation in Type II EC cell lines. (A, B)** Dose–response curves showing the effects of 96-hour HCI2509 or medroxyprogesterone 17-acetate (MPA) treatment on cell viability of **(A)** AN3CA and **(B)** KLE cells normalized to vehicle controls. EC50s and 95% CI’s were calculated using GraphPad Prism 6.0 and are reported where the R^2^ > 0.9. Data points are reported as mean and standard deviation (n = 3). **(C, D)** Proliferation (3 T5) assays showing cell doubling times for **(C)** AN3CA and **(D)** KLE cells with vehicle and increasing doses of HCI2509. Data points are reported as mean and standard deviation (n = 3). **(E, F)** Quantification of colonies formed by **(E)** AN3CA or **(F)** KLE cells in soft agar with either vehicle or HCI2509 treatment at varying concentrations. Error bars indicate SD of duplicate assays.

We first validated previous data suggesting that Type II endometrial carcinoma cells were sensitive to LSD1 inhibition with HCI2509 [35]. Both AN3CA and KLE cell lines exhibited a dose-dependent decrease in cell viability after 96 hours of treatment with HCI2509 (Figure 1A, B) with EC_50_ values determined at 499 nM and 435 nM, respectively (Figure 1A, B). In separate experiments, treatment with medroxyprogesterone 17-acetate (MPA) showed no effect on cell viability, confirming that both cell lines exhibit resistance to hormone treatment (Figure 1A, B). Having determined the EC_50_ we next tested the effect of HCI2509 on population doubling times using a 3T5 proliferation assay in treatment conditions below and above the EC_50_ (Figure 1C, D). HCI2509 decreased proliferation rates in a dose dependent manner in both AN3CA and KLE cell lines. Interestingly, even the lowest tested treatment concentration (0.3 X IC_50_) resulted in cytostasis in KLE cells. At and above the IC_50_, both cell lines exhibited negative growth, suggesting cell death.

In addition to the anti-proliferative effects seen in the 2-dimensional viability and proliferation assays, we also tested the ability of HCI2509 to impair anchorage-independent growth in soft agar. Cells were tested for colony formation at a range of concentrations spanning 30 nM to 10 μM. Based on the increased sensitivity of the KLE cells in the proliferation assay, the dose range tested in agars was shifted one half-log lower than that for AN3CA cells. HCI2509 impaired colony formation in both cells lines in a dose-dependent manner (Figure 1E, F). Above the viability EC_50_ for both cell lines, anchorage-independent growth was ablated, and at concentrations below the EC_50_ for KLE cells, colony formation was reduced, suggesting that HCI2509 impaired transformation at concentrations lower than those for which it induces cell death in KLE cells. AN3CA cells showed reduced colony formation near the viability EC_50_.

### LSD1 inhibition results in global histone methylation changes and induction of LSD1 target genes

**Figure 2:**
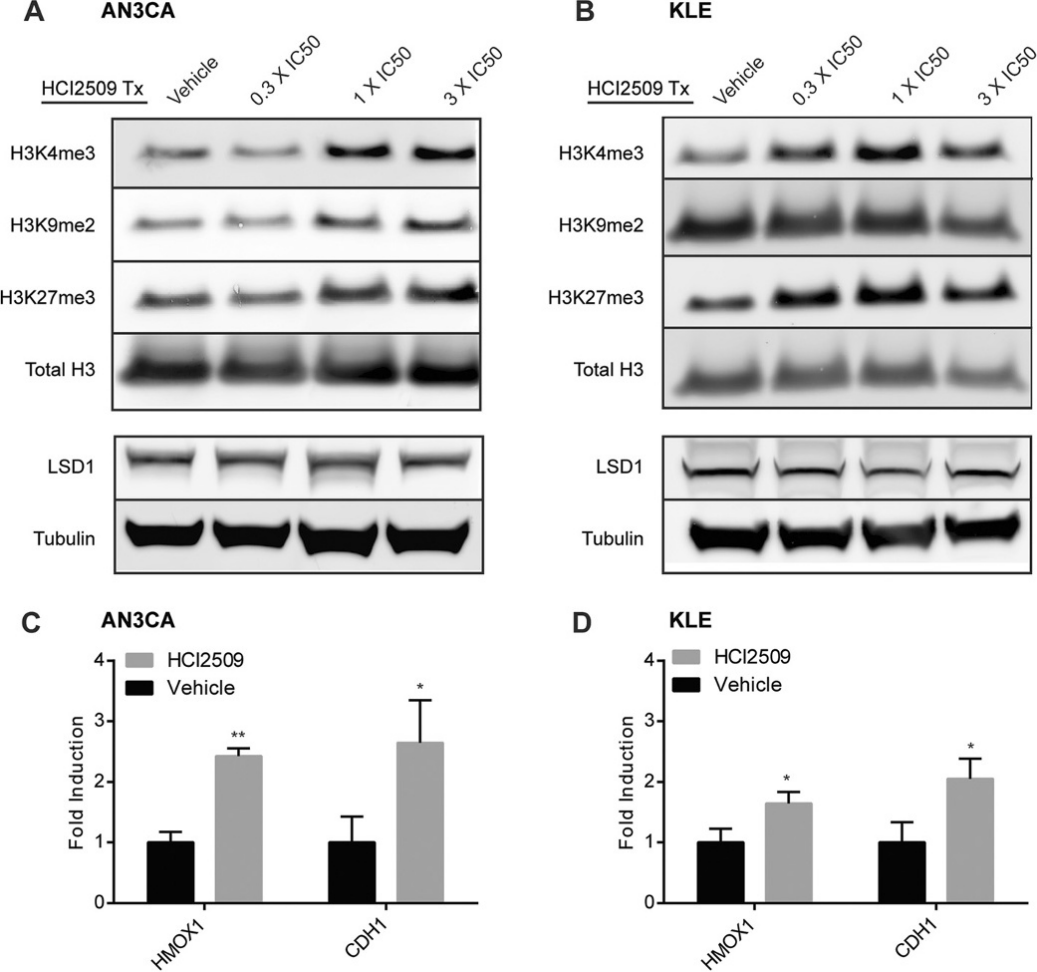
**Treatment with HCI2509 causes changes in global histone methylation and induces LSD1 target genes. (A, B)** Western blot analysis of H3K4me3, H3K9me2, H3K27me3 and LSD1 after 48 hours of vehicle or HCI2509 treatment at varying concentrations in **(A)** AN3CA and **(B)** KLE cells. Images are representative of two repeat experiments performed in triplicate. **(C, D)** qRT-PCR analysis of LSD1 target genes, *HMOX1* and *CDH1*, after treatment with 3X EC50 for **(C)** AN3CA and **(D)** KLE cells. Data represents the mean and standard deviation (n = 3) and all replicates were normalized to internal housekeeping gene *RPL19*.

LSD1 is the primary histone demethylase for the cell and having demonstrated dose-dependent effects on viability, proliferation, and transformation, we next investigated whether HCI2509 treatment also caused dose-dependent increases in histone methylation marks. We evaluated both LSD1 histone substrates, H3K4 and H3K9. Analysis of H3K4me1 and H3K4me2 showed no effect of HCI2509 treatment on the monomethyl mark and accumulation of H3K4me2 in only AN3CA cells (Additional file 1: Figure S2A, B). We next asked whether at 48 hours impaired demethylation of H3K4 may result in accumulation of the H3K4 trimethyl mark. While trimethyllysine is not chemically accessible to LSD1, the effect of demethylation at promoter H3K4 is gene repression, and impaired demethylation at that mark may result in increased levels of the transcriptionally activating H3K4me3 chromatin. Additionally, H3K4me3 is depleted in an LSD1-dependent fashion during the epithelial-to-mesenchymal transition (EMT) [41]. HCI2509 treatment resulted in a dose-dependent increase in H3K4me3 in both cell lines (Figure 2A, B).

In complex with the estrogen and androgen hormone receptors, LSD1 is shown to activate target gene expression through removal of repressive H3K9 methylation. H3K9me2 is also shown to be largely depleted during EMT through an LSD1-dependent mechanism and this loss of H3K9me2 is associated with transformation [41]. Thus, we evaluated the effects of HCI2509 on H3K9me2 and observed an increase in H3K9me2 in AN3CA cells (Figure 2A). Interestingly, treatment with HCI2509 showed no effect on H3K9me2 in KLE cells (Figure 2B). We also predicted that changes in global methylation status in either H3K4 or H3K9 would occur in synchrony with additional global changes to chromatin state, so we also blotted for H3K27me3, a mark typically associated with gene repression and heterochromatin [26]. HCI2509 treatment induced a dose-dependent increase in H3K27me3 in both cell lines. The observed elevation of histone methylation by HCI2509 occurred with no change observed for LSD1 protein levels (Figure 2A, B).

We also asked whether HCI2509 modulated expression of LSD1 target genes. Induction of *HMOX1* has been shown to be a biological readout for LSD1 engagement by HCI2509 [35, 38]. We additionally evaluated the expression of *CDH1* (E-cadherin). E-cadherin is a cell-surface adhesion molecule that is repressed during SNAIL-LSD1-mediated EMT and is often misregulated in Type II endometrial cancer [42, 43]. HCI2509 treatment induced increased transcription of both *HMOX1* and *CDH1* in both AN3CA and KLE cell lines (Figure 2C, D), suggesting LSD1 target engagement by HCI2509.

### LSD1 inhibition disrupts normal cell cycle progression in human endometrial cancer cell lines

**Figure 3:**
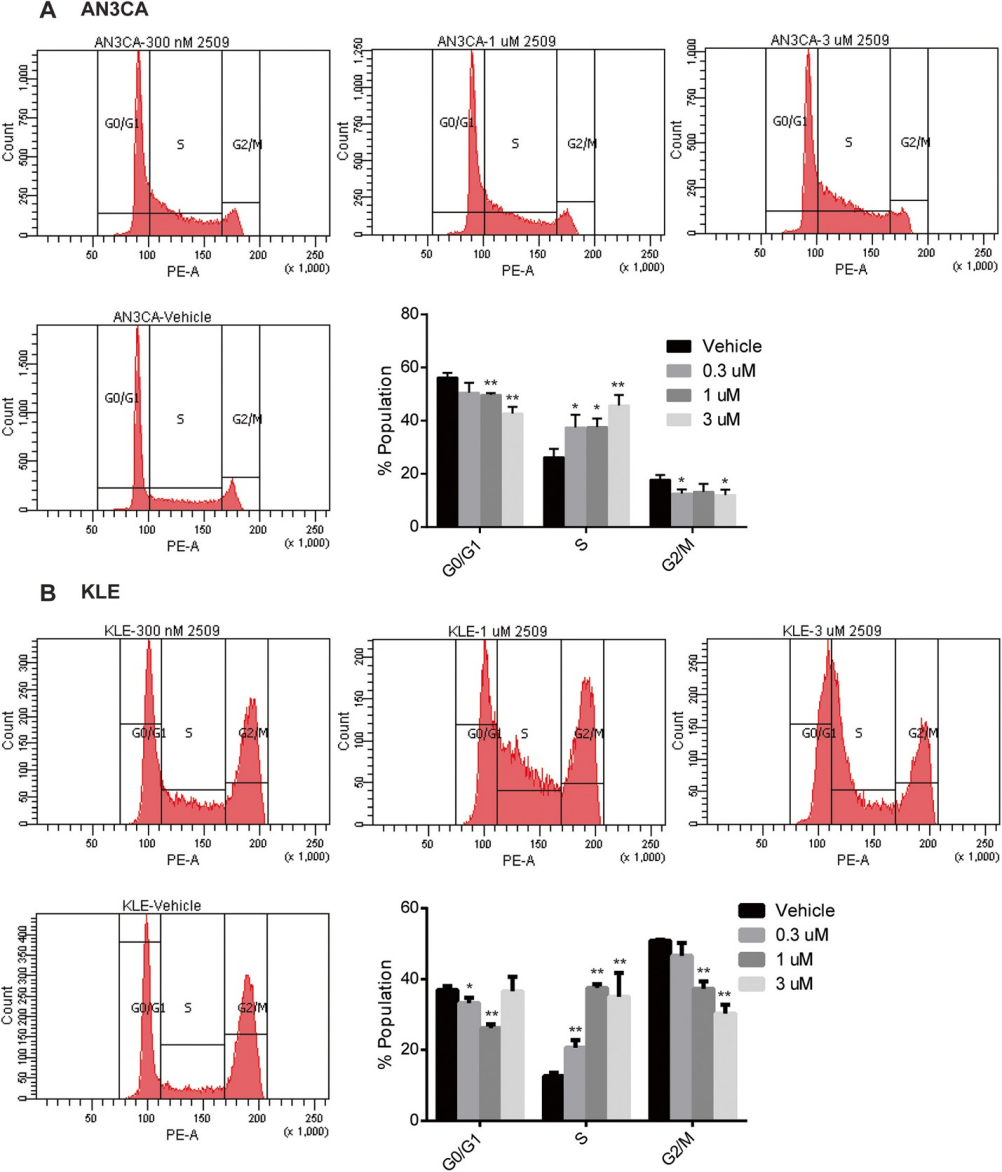
**Dose-dependent cell cycle perturbation in Type II EC cell lines with HCI2509 treatment. (A, B)** Cell cycle populations of **(A)** AN3CA and **(B)** KLE cell lines after exposure to vehicle, 300 nM, 1 μM, and 3 μM HCI2509 for 48 hours. 2 × 10^4^ counts and 1 × 10^4^ counts were used for AN3CA and KLE cells, respectively. Data is representative of four biological replicates. Mean and standard deviation are plotted (* p < 0.05, ** p < 0.01).

The observation of decreased proliferative rates prompted us to test the effect of HCI2509 treatment on cell cycle progression in both AN3CA and KLE cells. Cell cycle analysis was performed with either vehicle or HCI2509 exposure at 300 nM, 1 μM, or 3 μM for 48 hours. AN3CA cells showed a dose-dependent increase in the percentage of cells in S-phase (Figure 3A). This was accompanied by a decrease in the G0/G1 population. In a time-course experiment 3 μM HCI2509 shows an accumulation of cells in the G0/G1 population from 6–12 hours before developing the increased S-phase fraction at 24 and 48 hours (Additional file 2: Figure S3A). KLE cells show a similar accumulation in early S-phase with increasing concentrations of HCI2509 (Figure 3B). Unlike the AN3CA data, the increase in the S-phase fraction occurs at the expense of the G2/M population of cells. The time-course experiment with KLE cells in 3 μM HCI2509 interestingly never passed through the same distribution as observed for 1 μM HCI2509 at 48 hours, and failed to show any obvious change until 48 hours (Additional file 2: Figure S3B). These data suggest that LSD1 inhibition with HCI2509 perturbs cell cycle progression in both Type II endometrial carcinoma cell lines, most likely through an accumulation in early S-phase.

### HCI2509 induces apoptosis in AN3CA and KLE cells

**Figure 4:**
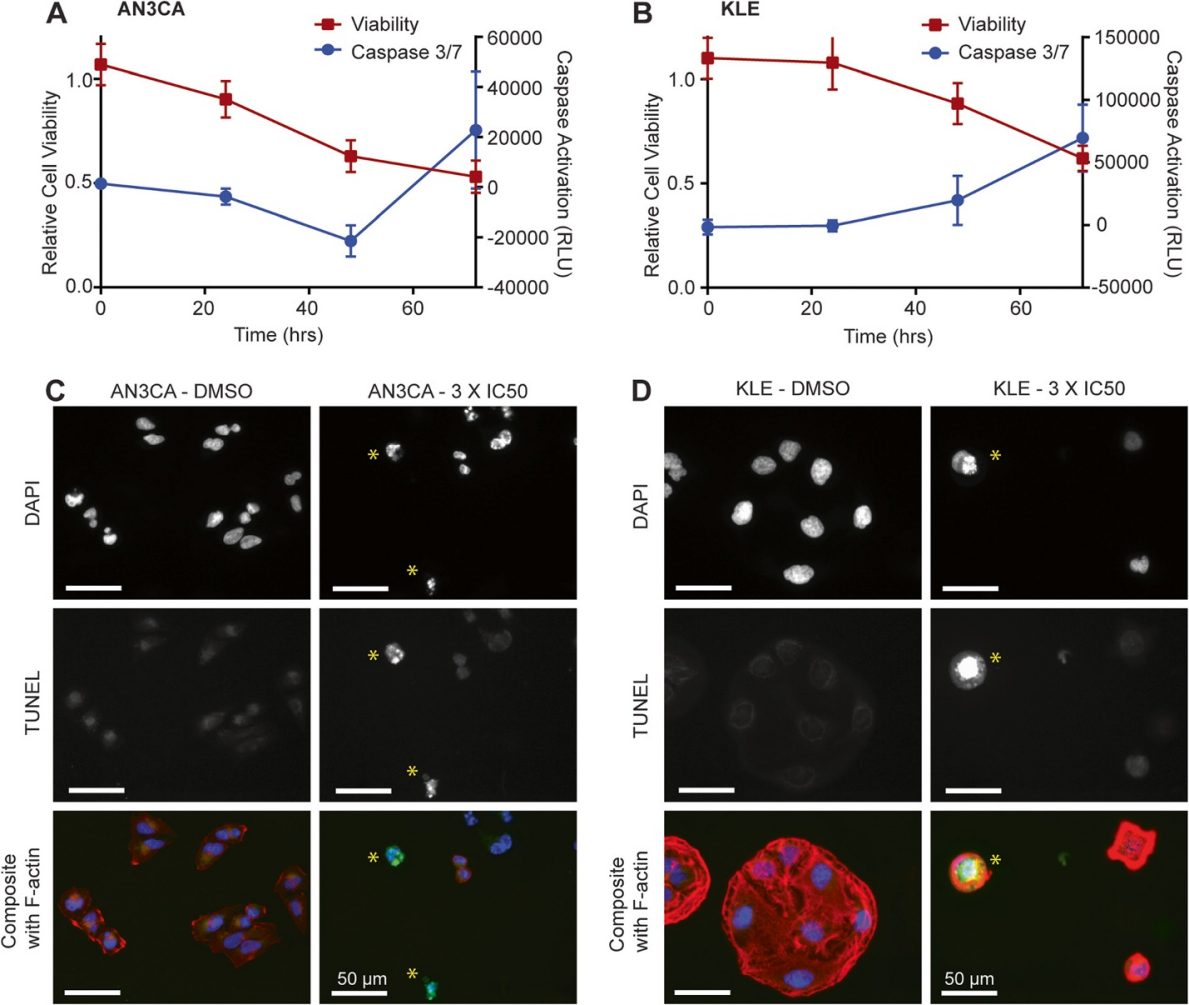
**HCI2509 induces apoptotic cell death. (A, B)** Cell viability and caspase activation at 0, 24, 48, and 72 hours in **(A)** AN3CA and **(B)** KLE cells treated with 3X EC50 HCI2509. Measurements were normalized to their respective vehicle (0.3% DMSO) sample at the appropriate time point. **(C, D)** Fluorescence microscopy images of **(C)** AN3CA and **(D)** KLE cell lines after exposure to either vehicle or 3X EC50 HCI2509 and then stained with TUNEL for apoptotic nuclei (green), DAPI for nuclei (blue), and phalloidin for actin (red). HCI2509 treatment induced apoptosis with apoptotic cells marked with (*).

In addition to cell cycle disruption, we investigated the mechanism causing negative cell doubling in both AN3CA and KLE cells. We hypothesized that HCI2509 treatment may cause apoptotic cell death and therefore tested both cell lines for caspase 3/7 activation. Caspase activity was assayed in parallel with cell viability using 3X the EC_50_ and comparing to vehicle control. Viability and caspase activation were assessed over a time-course of 72 hours in both cell lines. Interestingly, in the context of HCI2509 treatment AN3CA showed decreased cell viability and caspase activity over the course of 48 hours with increased caspase activation occurring at 72 hours (Figure 4A). The decrease in caspase activation during the first 48 hours of treatment is likely due to a decreased number of cells/well due to cytostatsis relative to vehicle. HCI2509-treated KLE cells showed a concomitant increase in caspase activity and decrease in cell viability over 72 hours (Figure 4B). These data suggest an initial cytostasis which is followed by apoptotic cell death induced after 48 hours. We next confirmed apoptotic cell death using fluorescent TUNEL staining. AN3CA and KLE cells were treated with either vehicle or 3X EC_50_ HCI2509 for 72 hours and then assayed for TUNEL staining. Both cell lines showed decreased cell density and the presence of apoptotic cells with HCI2509 treatment, while vehicle treated cells appeared healthy and well spread on the coverslip (Figure 4C, D, Additional file 3: Figure S4A, S4B). Internal controls for the TUNEL assay are reported in Additional file 3: Figure S4C. These results confirmed apoptotic cell death induced by HCI2509 treatment.

### HCI2509 leads to tumor regression in an orthotopic endometrial carcinoma mouse xenograft model

**Figure 5:**
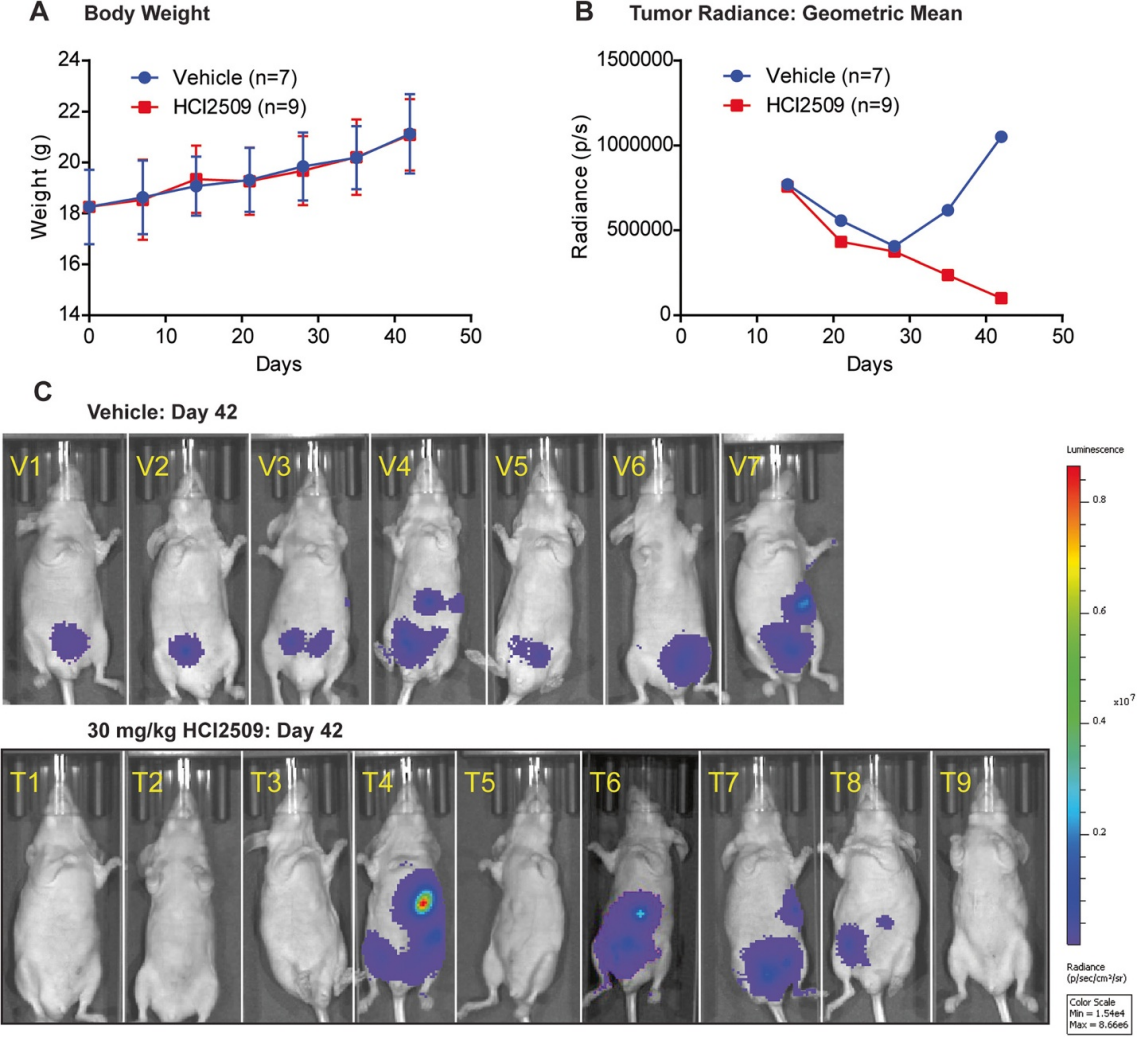
**HCI2509 treatment causes tumor regression** ***in vivo*** **. (A)** Average total body weight (g) of mice in both groups, vehicle and HCI2509 treatment, starting at implantation (day 0) through the course of the study. Data points shown represent the mean and standard deviation. **(B)** Quantified bioluminscence measurements of both the vehicle and HCI2509 treatment groups. Data is plotted as the geometric mean of total flux (photons/second). Daily treatment was initiated on day 7 (day 0 = implantation), such that day 14 represents the first day of imaging after the start of treatment. **(C)** Individual mouse images from study day 42 (day 35 of treatment). All images are on the same luminescence scale from 1.54 × 10^4^ p/s to 8.66 × 10^6^ p/s. Note: Animal #6 in the treatment group was sacrificed on study day 36, therefore the image is from study day 35 imaging.

We further evaluated the efficacy of HCI2509 in an orthotopic xenograft model of endometrial carcinoma utilizing the KLE cell line stably transfected with luciferase to facilitate bioluminescence imaging. After implantation (day 0) and recovery, bioluminescence was measured weekly for the duration of the study (42 d). Total body weight was measured 3 times weekly, and weekly points were plotted (Figure 5A). At day 7, animals with detectable tumor were randomized into vehicle only and HCI2509 treatment groups (Additional file 4: Figure S5A). We observed the tumor luminescence values were better fit to a log-normal distribution than a normal distribution, which is common for various biological phenomena such as latency times for infections or survival times after a diagnosis of cancer (Additional file 4: Figure S5B) [44]. For this reason, the geometric mean of the tumor volumes for both conditions are plotted (Figure 5B). Values observed at day 7 were higher than those observed for the remainder of the study and therefore excluded from the graph. This initial burst of proliferation, and associated luminescence, followed by a drop off before later hitting exponential growth is commonly observed in xenograft studies. After 35 days of treatment (day 42 of the study) proliferating disease was observed in all of the vehicle treated animals, while 5 of the 9 drug treated animals showed no detectable luminescence (Figure 5C). Lack of luminescence is incorporated as the background reading of the instrument for each day of the experiment, as determined by an unimplanted, non-tumor bearing, healthy control. We used a Fisher’s exact test to evaluate the effect of treatment vs. vehicle on either tumor or regression and found HCI2509 significantly associated with tumor regression (p = 0.034). No difference in body weight was seen between the vehicle and treatment groups indicating tolerability of HCI2509. The luminescence readout for the untreated control group are plotted together with data from the vehicle and treatment groups in Additional file 4: Figure S5D as are the body weight measurements including the non-tumor bearing control. When considered with the *in vitro* data suggesting decreased proliferation, transformation and induced apoptosis in concert with increased global histone methylation and LSD1 engagement, these data support LSD1 inhibition with HCI2509 as a potential therapeutic strategy for Type II endometrial carcinoma.

## Discussion

LSD1 is an emerging target for poorly differentiated and aggressive solid malignancies. Our findings suggest that LSD1 inhibition holds potential as a new therapeutic strategy for Type II endometrial cancer, which may accompany current state of the art treatment of EC in the future. Targeted LSD1 inhibition with HCI2509 showed potent anti-cancer activity both *in vitro* and *in vivo* with multiple tumor regressions observed in our orthotopic EC model. Type II EC constitutes an unmet medical need, with disproportionately high number of annual EC deaths relative to the proportion of Type II EC diagnoses as compared to Type I disease. While it is known that epigenetics, genetics, and the environment all contribute to the development of EC, recent studies demonstrating mutations in chromatin remodeling complexes as driver events in Type II EC [15–17] underscore the need for research to evaluate more effective and new therapeutic strategies targeting these mechanisms.

Chromatin modifiers or ubiquitin ligase complexes were recently implicated in 35% of clear cell endometrial and 50% of serous endometrial tumors [15]. One of the most commonly altered genes was *CHD4*, a member of the NuRD complex, along with the observation of frequent mutations in *MBD3*, another NuRD component [15]. *CHD4* mutations were all predicted to disrupt normal function of the protein, suggesting a functional role in the development of EC [15]. LSD1 is bound by NuRD and has been shown to repress both tumor suppressor genes [45] and genes associated with metastasis and invasion [46] in complex with NuRD. It is possible that the role of LSD1 is altered in endometrial cancer through functional mutations in NuRD members, and this results in sensitivity to LSD1 inhibition. However, the role of NuRD mutations in endometrial cancer remain unstudied. Detailed studies addressing the role of NuRD and whether LSD1 and NuRD work in concert in endometrial cancer could lead to insight regarding which patients may benefit from LSD1 inhibition or other epigenetic intervention.

This is especially true in light of the results showing that not only are LSD1 substrates affected by HCI2509, H3K27me3 was elevated in both cell lines, suggesting LSD1 inhibition exhibited downstream epignomic regulatory effects. Interestingly, decreased H3K27me3 is associated with poorer survival in both breast and ovarian cancers [47], though this has not been studied in Type II EC. The H3K27 methyltransferase EZH2 is overexpressed in ~60% of Type II EC and has been linked to focal adhesion kinase (FAK) and deregulation of E-cadherin [13]. This presents another possible avenue to define the functional linkage of epigenetic misregulation with Type II EC biology. The differences observed between cell lines with respect to H3K9me2 are consistent with the highly contextual dependence of LSD1 function. In summary, the histone methylation data presented here contrasts with that shown for HCI2509 in Ewing sarcoma [38], and emphasizes the importance of additional mechanistic studies to be conducted in the future to better define LSD1 biology.

Corroborating other *in vitro* findings, results observed for the effects of HCI2509 on the cell cycle showed a dose-dependent increase in S-phase and decrease in the G2/M for both AN3CA and KLE cells. Time course analysis revealed what appeared to be a moderate G1/G0 arrest at 12 hours in AN3CA cells, though not in the KLE cells. The primary effect seen was an accumulation in early S-phase in both cell lines. LSD1 has been shown to play a critical role in maintaining the cell cycle in embryonic stem cells [48, 49], as well as promoting proliferation and cell cycle progression in cancer cells [50, 51], and the data here is consistent with this observation.

One of the biggest limitations in studying new epigenetic therapies in a given disease is the lack of mechanistic understanding to distinguish which molecular events are drivers and passengers in tumorigenesis. In the meantime, translational progress requires potent and specific tool compounds and to validate new therapeutic strategies. Because epigenetics represents the intersection between genes and the environment, it is likely that the phenomena observed in tissue culture will not represent the disease state in a mouse, and further, the difficulties translating from mouse studies to humans is well documented [52]. To mitigate these issues, we placed emphasis on early testing of whether epigenetic modulation with an LSD1 inhibitor would work *in vivo* in Type II EC. Further, we also wanted to recapitulate the tumor environment as reliably as possible in an orthotopic setting using relevant human cancer cells. In our KLE model, the generated tumors showed a dip in luminescent signal after the first week as is common and in the vehicle group signal rebounded in an exponential growth pattern by day 42. The doubling time for KLE cells in tissue culture is fairly slow, around 72 hours, indicating *in vivo* disease progression rate being consistent with the character of the cell line. We were encouraged to see signal present throughout the abdominal cavity in several mice throughout the study, as this suggested an invasive and disseminated disease. Based on the limited number of animals in this pilot *in vivo* study, we favored endpoints over additional tissue evaluation of responsive tumors to better understand molecular effects caused by HCI2509 treatment, rendering responsive tumors unavailable for additional experiments. Further dose finding, frequency, and survival studies are planned.

Ultimately, this is the first data including histone methylation changes, target gene elevation, and induced apoptosis in EC and is very encouraging. Additional studies should evaluate LSD1 inhibition in more translational and patient-derived models, both *in vitro* and *in vivo*. To do so will require expanding the mechanistic insight based on the recent implication of chromatin remodelers in Type II EC using more potent and specific tool compounds. Additional investigation of epigenetics, as well as the relationship between specific pharmacodynamic and pharmacokinetic markers of response, will be needed to gain an in depth understanding of these mechanisms in the development of EC. Furthermore, LSD1 inhibition with HCI2509 should be evaluated for synergistic effects with other targeted inhibitors of other pathways implicated in Type II EC, such as FAK [13] signaling, as well as conventional treatment modalities including hormone therapy currently applied in the treatment of EC.

## Conclusions

In conclusion, we have demonstrated that the treatment of Type II endometrial carcinoma cell lines with the LSD1 inhibitor HCI2509 decreased proliferation and transformation, induced histone methylation and LSD1 target gene expression, perturbed cell cycle progression, and induced apoptotic cell death *in vitro*. Moreover, in an orthotopic endometrial carcinoma animal model with human KLE cells, HCI2509 treatment resulted in 5/9 tumor regressions over the course of 42 days. Taken together these findings support further investigation of the role of LSD1 in Type II endometrial carcinoma biology as well as LSD1 inhibition as a novel therapeutic strategy for this aggressive gynecologic malignancy.

## Authors’ information

Sunil Sharma and Margit Janat-Amsbury are co-senior authors.

## Electronic supplementary material

Additional file 1: Figure S2: Changes to histone H3 lysine 4 monomethyl and dimethyl marks with HCI2509 treatment. (A, B) Western blot analysis of H3K4me1 and H3K4me2 after 48 hours of vehicle or HCI2509 treatment at varying concentrations in (A) AN3CA and (B) KLE cells. Images are representative of two repeat experiments performed in triplicate. (PDF 49 KB)

Additional file 2: Figure S3: Time course evaluation of cell cycle perturbations caused by HCI2509 treatment. (A, B) Cell cycle populations of (A) AN3CA and (B) KLE cell lines after exposure to vehicle (0 and 48 hours) or 3 μM HCI2509 (6, 12, 24, and 48 hours). 2 × 10^4^ counts and 1 × 10^4^ counts were used for AN3CA and KLE cells, respectively. Data is representative of four biological replicates. (PDF 554 KB)

Additional file 3: Figure S4: TUNEL assay replicates and controls. (A, B) Fluorescence microscopy images of (A) AN3CA and (B) KLE cell lines after exposure to either vehicle or 3X EC50 HCI2509 and then stained with TUNEL for apoptotic nuclei (green), DAPI for nuclei (blue), and phalloidin for actin (red). HCI2509 treatment induced apoptosis with apoptotic cells marked with (*). (C) Fluorescence microscopy images of TUNEL negative and positive controls with untreated AN3CA and KLE cells. Negative controls were generated by adding labeled nucleotide with no enzyme and positive controls were generated by pretreating DNase before TUNEL labeling. Cells are stained with TUNEL (green), DAPI (blue), and phalloidin for actin (red). (PDF 194 KB)

Additional file 4: Figure S5: HCI2509 treatment causes tumor regression *in vivo*. (A) Individual mouse images from study day 7 (day 0 of treatment). All images are on the same luminescence scale from 1.54 × 10^4^ p/s to 8.66 x 10^6^ p/s. (B) Quantified bioluminscence measurements of both the vehicle and HCI2509 treatment groups pooled. Total flux (photons/second) was rank ordered and plotted on a semi-log plot. The linearity of the log-transformed data supports a log-normal distribution. (C) Fisher’s exact test shows significant association of HCI2509 treatment with tumor regression. Both the observed and expected contingency tables are shown with the reported p-value. (D) Tumor volume and body weight measurements including both the untreated and unimplanted control. Tumor volumes are plotted as the geometric mean of the observed luminescent signal and body weight is plotted as the average and SD. (PDF 150 KB)

## Abbreviations

APC: Adenomatous polyposis coli
ARID1A: AT rich interactive domain 1A
CD133: Cluster of differentiation 133
CDH1: E-cadherin
EC: Endometrial carcinoma
EC50: Effective concentration at 50%
EMT: Epithelial-to-mesenchymal transition
EP300: E1A binding protein p300
EZH2: Enhancer of zeste homolog 2
FAK: Focal adhesion kinase
FGFR: Fibroblast growth factor receptor
H3: Histone H3
H3K4 (me3): Histone H3 lysine 4 (dimethylated)
H3K9 (me2): Histone H3 lysine 9 (trimethylated)
H3K27 (me3): Histone H3 lysine 27 (trimethylated)
HMOX1: Heme oxygenase 1
LSD1: Lysine-specific demethylase 1
MGMT: O-6-methylguanine-DNA methyltransferase
MLH1: mutL homolog 1
MPA: Medroxyprogesterone 17-acetate
NuRD: Nucleosome remodeling and deacetylase
PTEN: Phosphatase and tensin homolog
TAF1: TATA box binding protein (TBP)-associated factor
TUNEL: Terminal deoxynucleotidyl transferase dUTP nick end labeling.
