# Supplementary figures and images for: Reversible inhibition of lysine specific demethylase 1 is a novel anti-tumor strategy for poorly differentiated endometrial carcinoma
Source: BMC Cancer. 2014 Oct 9;14:752. doi: 10.1186/1471-2407-14-752 (PMC4197342; doi:10.1186/1471-2407-14-752)

**Figure S2**

**A**

**AN3CA**

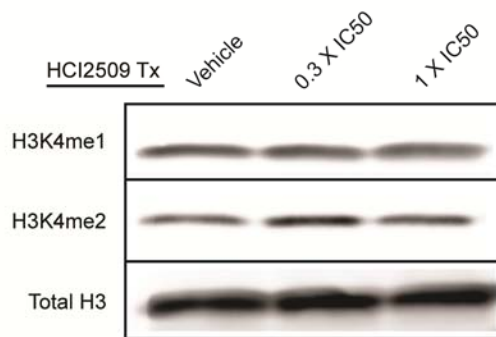

**B**

**KLE**

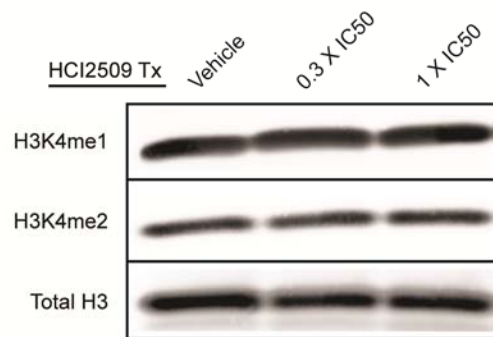

Supplement: Supplementary file 1 — Additional file 1: Figure S2: Changes to histone H3 lysine 4 monomethyl and dimethyl marks with HCI2509 treatment. (A, B) Western blot analysis of H3K4me1 and H3K4me2 after 48 hours of vehicle or HCI2509 treatment at varying concentrations in (A) AN3CA and (B) KLE cells. Images are representative of two repeat experiments performed in triplicate. (PDF 49 KB) [file 12885_2014_4932_MOESM1_ESM.pdf]

**Figure S3**

**A**

**AN3CA**

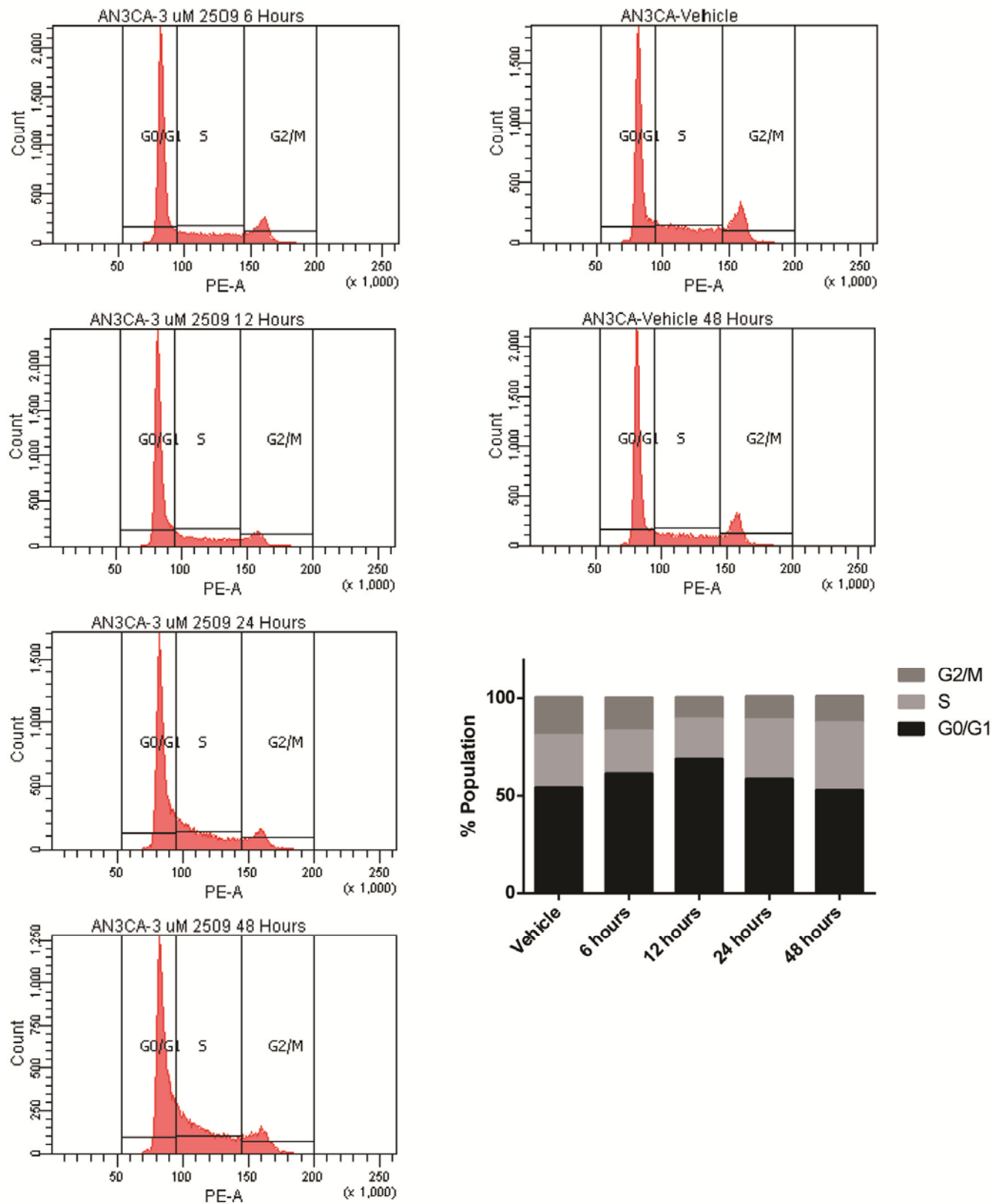

Figure S3

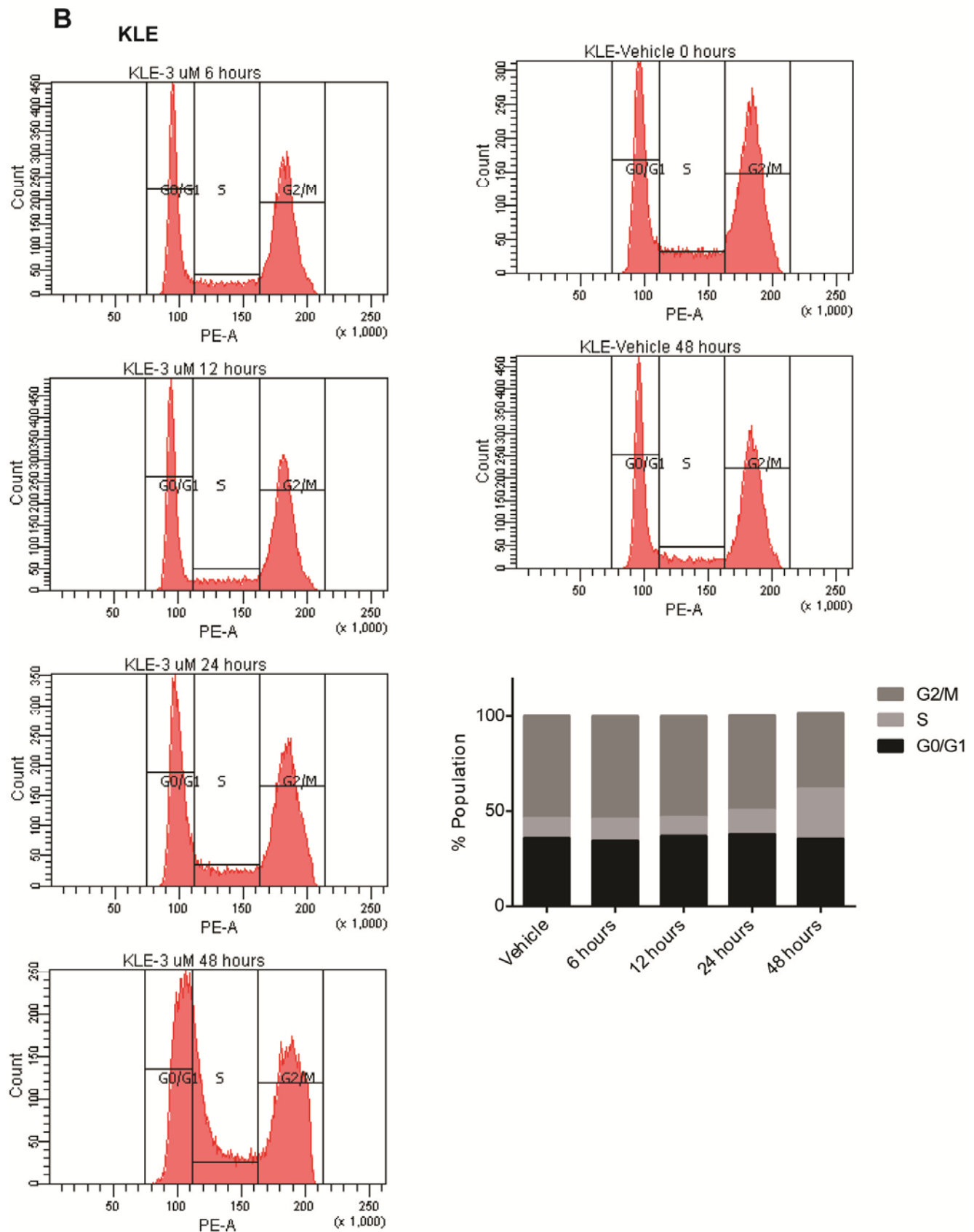

Supplement: Supplementary file 2 — Additional file 2: Figure S3: Time course evaluation of cell cycle perturbations caused by HCI2509 treatment. (A, B) Cell cycle populations of (A) AN3CA and (B) KLE cell lines after exposure to vehicle (0 and 48 hours) or 3 μM HCI2509 (6, 12, 24, and 48 hours). 2 × 10^4^ counts and 1 × 10^4^ counts were used for AN3CA and KLE cells, respectively. Data is representative of four biological replicates. (PDF 554 KB) [file 12885_2014_4932_MOESM2_ESM.pdf]

**Figure S4**

**A**

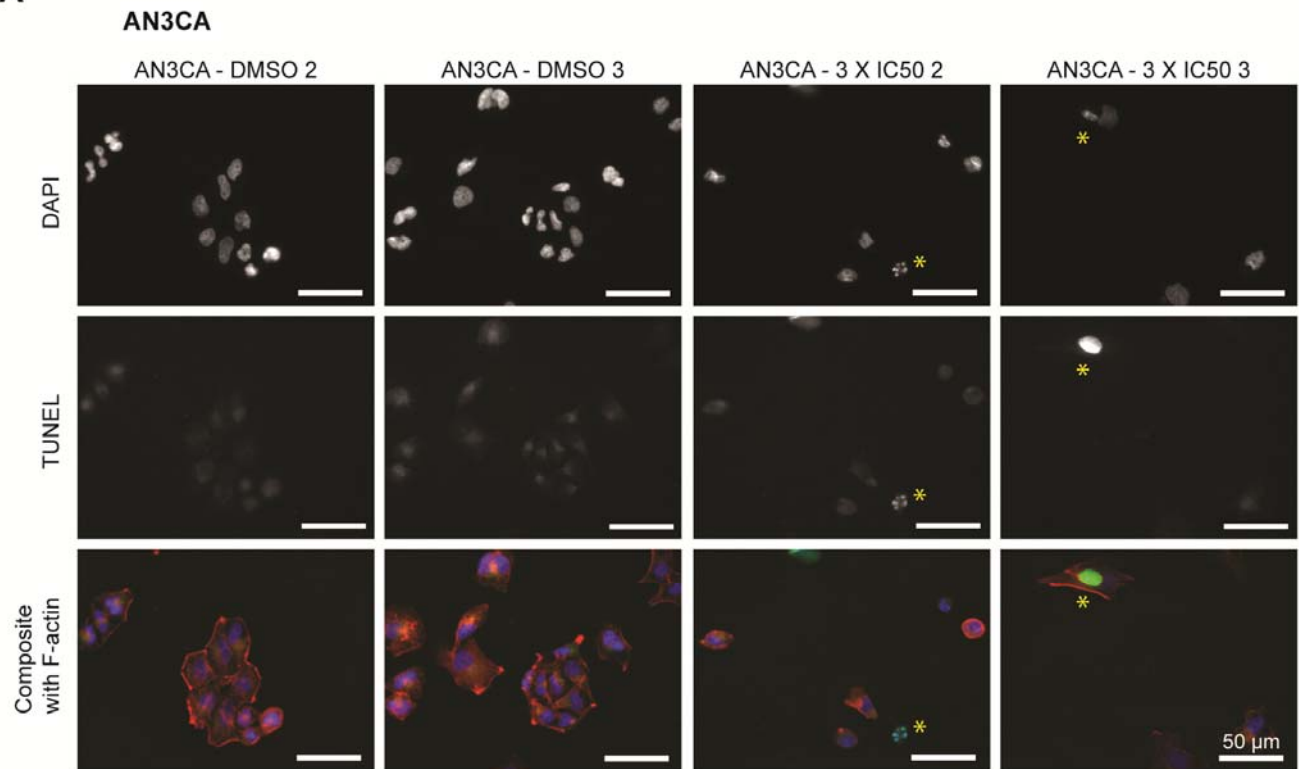

**B**

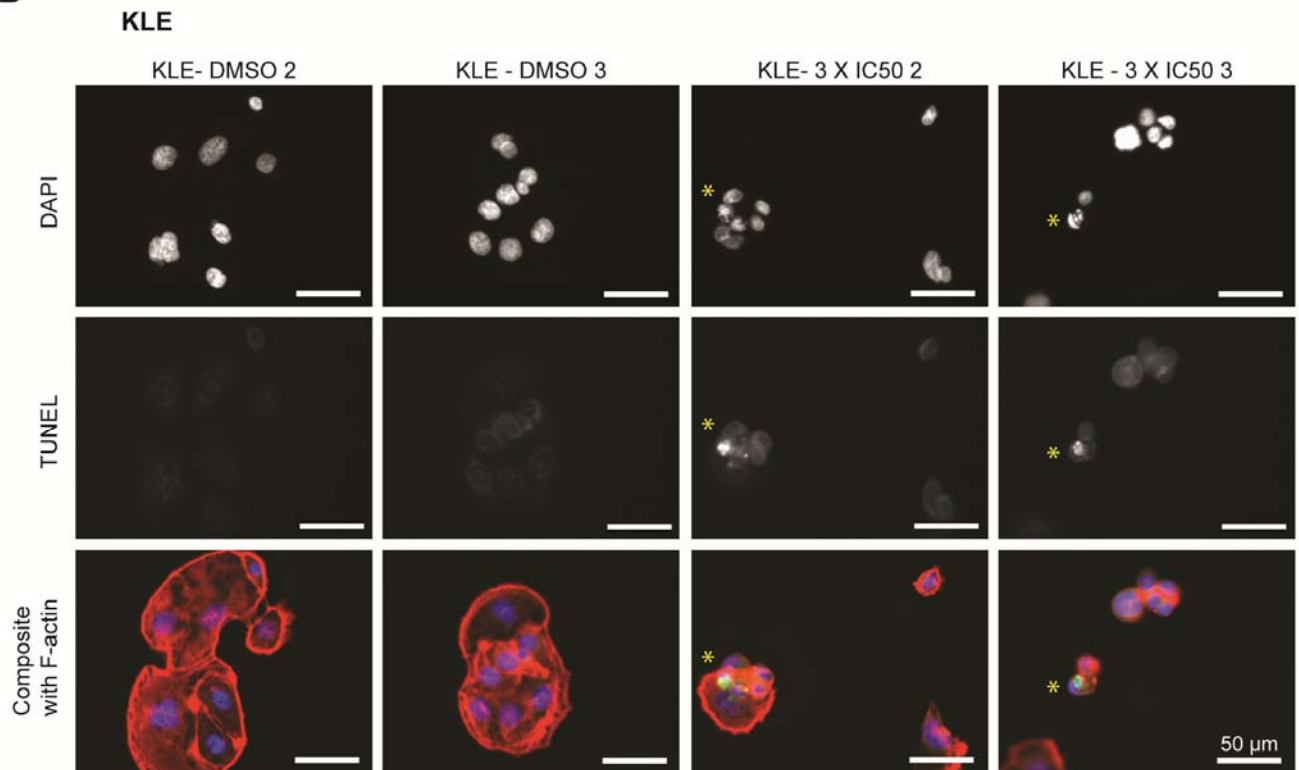

Figure S4

C

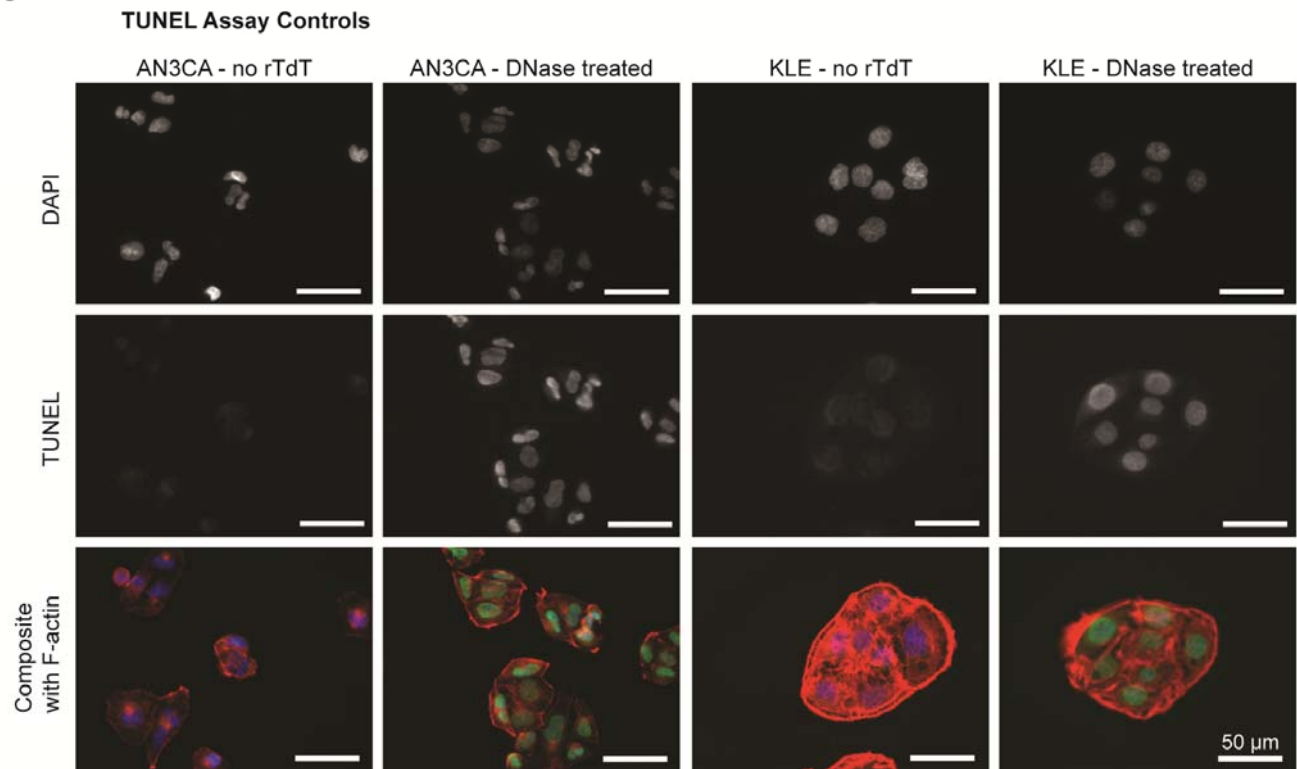

Supplement: Supplementary file 3 — Additional file 3: Figure S4: TUNEL assay replicates and controls. (A, B) Fluorescence microscopy images of (A) AN3CA and (B) KLE cell lines after exposure to either vehicle or 3X EC50 HCI2509 and then stained with TUNEL for apoptotic nuclei (green), DAPI for nuclei (blue), and phalloidin for actin (red). HCI2509 treatment induced apoptosis with apoptotic cells marked with (*). (C) Fluorescence microscopy images of TUNEL negative and positive controls with untreated AN3CA and KLE cells. Negative controls were generated by adding labeled nucleotide with no enzyme and positive controls were generated by pretreating DNase before TUNEL labeling. Cells are stained with TUNEL (green), DAPI (blue), and phalloidin for actin (red). (PDF 194 KB) [file 12885_2014_4932_MOESM3_ESM.pdf]
