# Supplementary material for: Reversible inhibition of lysine specific demethylase 1 is a novel anti-tumor strategy for poorly differentiated endometrial carcinoma
Source: BMC Cancer. 2014 Oct 9;14:752. doi: 10.1186/1471-2407-14-752 (PMC4197342; doi:10.1186/1471-2407-14-752)

Figure S5

A

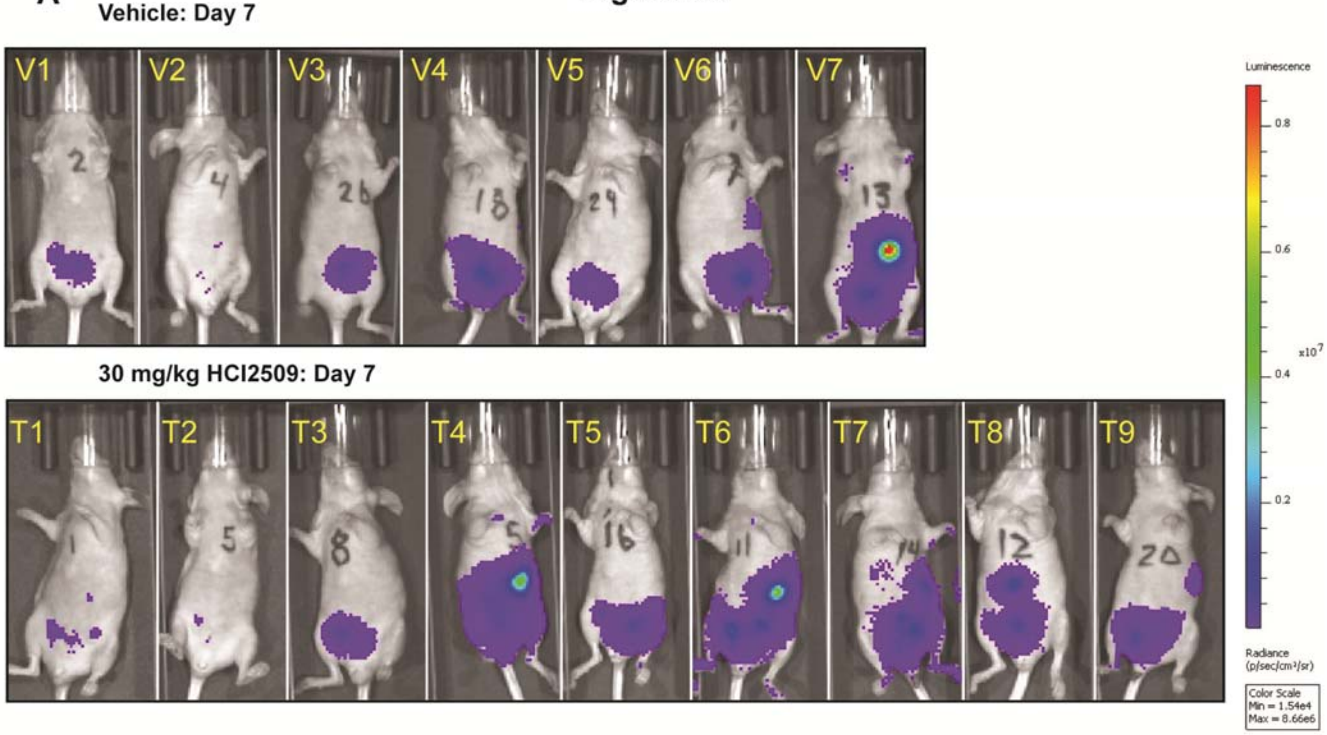

B

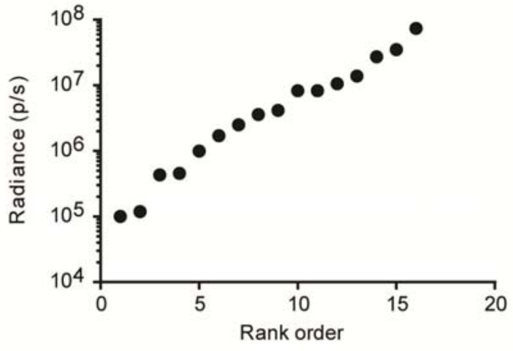

C

Fisher's exact test

Observed

	Tumor	Regression	
Vehicle	7	0	7
HCl2509	4	5	9
	11	5	16

$p = 0.034$

Expected

	Tumor	Regression	
Vehicle	5	2	7
HCl2509	6	3	9
	11	5	16

D

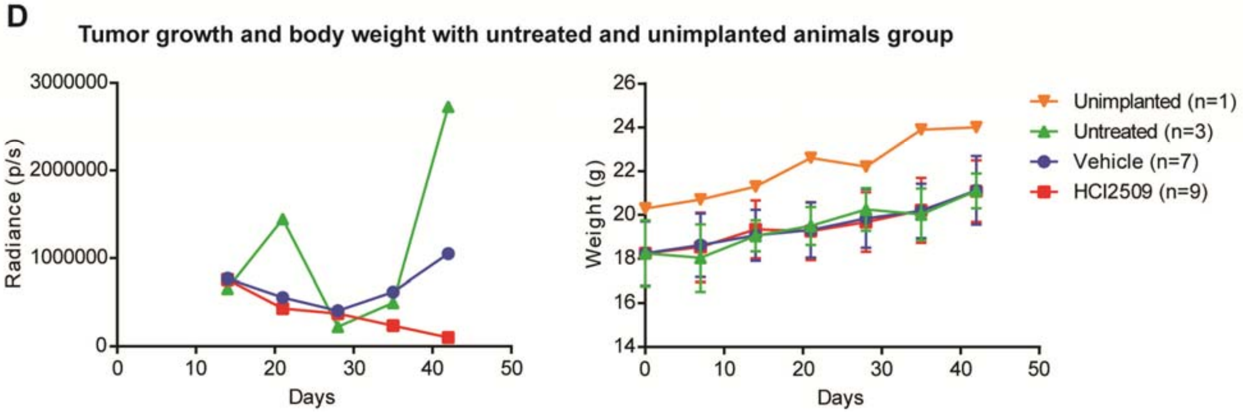

Supplement: Supplementary file 4 — Additional file 4: Figure S5: HCI2509 treatment causes tumor regression in vivo. (A) Individual mouse images from study day 7 (day 0 of treatment). All images are on the same luminescence scale from 1.54 × 10^4^ p/s to 8.66 x 10^6^ p/s. (B) Quantified bioluminscence measurements of both the vehicle and HCI2509 treatment groups pooled. Total flux (photons/second) was rank ordered and plotted on a semi-log plot. The linearity of the log-transformed data supports a log-normal distribution. (C) Fisher’s exact test shows significant association of HCI2509 treatment with tumor regression. Both the observed and expected contingency tables are shown with the reported p-value. (D) Tumor volume and body weight measurements including both the untreated and unimplanted control. Tumor volumes are plotted as the geometric mean of the observed luminescent signal and body weight is plotted as the average and SD. (PDF 150 KB) [file 12885_2014_4932_MOESM4_ESM.pdf]
